# Size Does Not Make the Difference: 3D/4D Transperineal Sonographic Measurements of the Female Urethra in the Assessment of Urinary Incontinence Subtypes

**DOI:** 10.1155/2016/1810352

**Published:** 2016-11-21

**Authors:** Tomas Kupec, Ulrich Pecks, Charlotte M. Gräf, Elmar Stickeler, Ivo Meinhold-Heerlein, Laila Najjari

**Affiliations:** ^1^Clinic for Gynaecology and Obstetrics, University Hospital RWTH Aachen, Pauwelsstraße 30, 52074 Aachen, Germany; ^2^Clinic for Gynaecology and Obstetrics, University Hospital Schleswig-Holstein, Arnold-Heller-Straße 3, 24105 Kiel, Germany

## Abstract

*Purpose.* The objective was to evaluate the usefulness of transperineal ultrasound in the assessment of the urethral length and urethral lumen by 3D/4D transperineal sonography to discriminate between female patients with subtypes of urinary incontinence.* Methods.* A total of 150 female patients underwent an examination because of urinary incontinence. 41 patients were diagnosed with urgency urinary incontinence (OAB), 67 patients were diagnosed with stress urinary incontinence (SUI), and 42 patients were in the control group (CTRL). Three diameters of the urethral lumen (proximal (U1), medial (U2), and distal (U3)) and the urethral length were measured. By the assessment of the urethral lumen, the presence of the urethral funneling was evaluated.* Results.* We found a significant difference in the urethral length and urethral lumen U2 of OAB and SUI versus CTRL. The urethral length was significantly greater (*P* < 0.05) and the urethral lumen was significantly wider (*P* < 0.05) in the patients with urinary incontinence. The incidence of the urethral funneling was significantly higher (*P* < 0.05) in the study groups with urinary incontinence than in the control group.* Conclusions.* Our results have shown the urethral changes obtained by ultrasound in patients with urinary incontinence, but they are still insufficient to distinguish between subtypes of urinary incontinence.

## 1. Introduction

The female urethra is functionally and anatomically a complex tubular organ extending below the bladder. Its crucial functional role is to maintain continence during bladder filling and to allow emptying during the voiding phase. One of the mechanisms involved in controlling continence is the urethral tonus. The urethral tonus is provided by the urethral smooth muscles, the urethral striated muscle, and the vascular elements within the submucosa [1]. Striated external urethral sphincter (rhabdosphincter) encircles the urethra in its middle part. It is responsible for increasing intraurethral pressure during times of need and contributes by about one-third of the resting tone of the urethra. The urethral smooth muscle blockade additionally reduces resting urethral closure pressure by about one-third. Lastly, the urethral submucosa with its prominent vasculature is partly responsible for the urethral closure. Occlusion of arterial flow to the urethra decreases resting urethral closure pressure [2].

It is possible to visualise the parts of the urethra with different echogenicity by the performance of transperineal ultrasound: the outer layer with external striated muscle, the middle layer with smooth muscle, the inner layer, which corresponds to the connective tissue with the vessels and submucosa, and the central part which represents the urethral lumen and the mucosa. Ultrasound based clinical examination became increasingly important. New generation high-resolution ultrasound offers the advantages of visualising anatomical structures and in the same time allows for functional assessment. Moreover, it is a noninvasive and cheap technique without any ionising radiation. 3D/4D ultrasound additionally allows virtual reconstruction of the urethra for precise evaluation and hence offers a technique with excellent intra- and interobserver repeatability [3, 4].

Changes in anatomical structures which provide urethral tonus can be assessed by ultrasound and were described for patients with stress urinary incontinence in previous studies. This includes a thinner striated external urethral sphincter as well as thinner urethral smooth muscle [5] and changes in the urethral vasculature [6, 7]. As a general doctrine, it has been proposed repeatedly that urethral length is shorter in patients with stress urinary incontinence [8, 9] though evidence from properly structured clinical trials is low. However, as stress urinary incontinence is a result of relaxation of the periurethral encircling connective tissue, elongation or enlargement of the urethra rather than shortening is the more likely consequence. Hence, we hypothesized that urethral length is greater and urethral lumen is wider in patients with stress urinary incontinence than in healthy controls or patients with urgency urinary incontinence.

The aim of this study was to evaluate the usefulness of transperineal ultrasound in the assessment of the urethral length and urethral lumen by 3D/4D sonography to discriminate between patients with subtypes of urinary incontinence. We aimed, by this new diagnostic approach, at providing a basis for future selection of patients that may benefit from specific treatment strategies. We assigned this study to the development stage of the IDEAL method (Stage 2a) [10].

## 2. Materials and Methods

A total of 150 female patients were included in our retrospective study. The women underwent an examination in the outpatient department of the urogynaecology centre at the University Hospital RWTH Aachen, because of urinary incontinence. The data were collected between 2009 and 2015. The diagnosis was based on anamnesis, including ICIQ-SF (International Consultation on Incontinence Questionnaire-Short Form), gynaecological examination, stress test, transperineal ultrasound, and urodynamic testing.

A single experienced senior physician performed the urodynamic testing. The examination was based on the ICS criteria [11].

**Figure 1:**
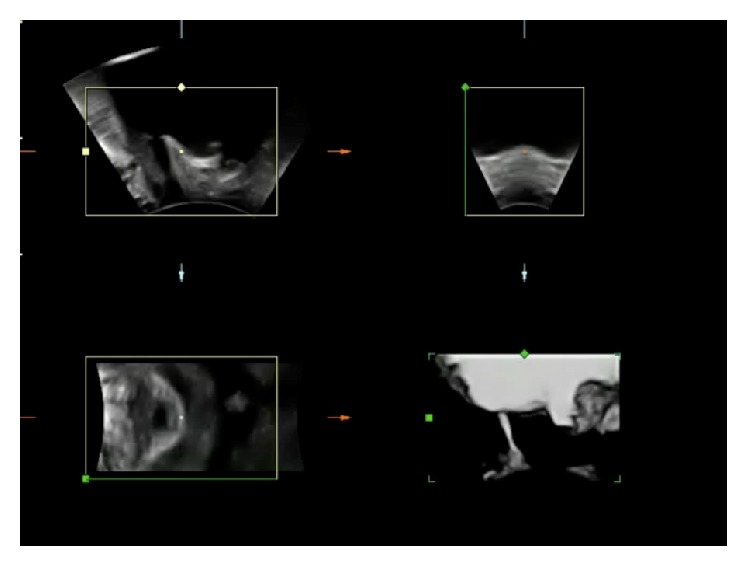
3D/4D transperineal ultrasound image.

Transperineal ultrasound was performed by a single experienced senior physician qualified according to the DEGUM level II standard (German Society for Ultrasound in Medicine). The ultrasound examination was performed under the same standard condition. Briefly, the patient was lying during the examination on the exam chair in the supine position. Bladder filling volume was approximately 300 mL. The ultrasound system (GE Medical Systems, Zipf, Austria; Voluson 730 Expert, E8) with a perineal ultrasound transducer (frequency range: 3.5–5 MHz) was used for all examinations. The ultrasound transducer was placed on the perineum with a beam angle of 70°. To avoid the compression of the urethra, the pressure on the transducer was produced as low as possible. A 3D/4D simultaneous view of multiple parallel slices in real time (Figure 1) was used for better assessment of the position and mobility of the urethra. The acquisition time for one volume data set was 5–7 s. During this period of time, we were imaging the bladder with bladder neck and bladder base in the longitudinal plane, the urethra in the largest diameter, and the transverse plane of the pubic symphysis with ligamentum arcuatum, pubic bone, and interpubic disc. The examination was performed in a state of relaxation, by contraction of the pelvic floor, by Valsalva manoeuvre, and during coughing. The process of the examination was recorded and saved on hard disk.

For analysis, the clinic database was searched for patients with the diagnosis of de novo urgency urinary incontinence (OAB) and stress urinary incontinence (SUI). The diagnosis was based on the IUGA/ICS criteria [12]. All patients with SUI complained of activity related incontinence, which was also presented by evaluation of ICIQ-SF. Stress test was positive and urodynamic testing confirmed the diagnosis of SUI. All patients with OAB complained of involuntary loss of urine associated with urgency, which was confirmed by filling of ICIQ-SF. Stress test was negative and the urodynamic testing confirmed the diagnosis of OAB. Exclusion criteria in the group of patients with urinary incontinence were the diagnosis of mixed urinary incontinence, previous urogynaecological surgery or other invasive urogynaecological therapies, pelvic radiation, and descent of the anterior vaginal wall, posterior vaginal wall, the uterus, or the apex of the vagina (POP-Q II or more).

Control group (CTRL) was chosen from patients, who were examined in our department during the same time period without symptoms of urinary incontinence. The CTRL did not suffer on pelvic organ prolapse (POP-Q II or more) and did not undergo previous urogynaecological surgery or other invasive urogynaecological therapies and pelvic radiation.

**Figure 2:**
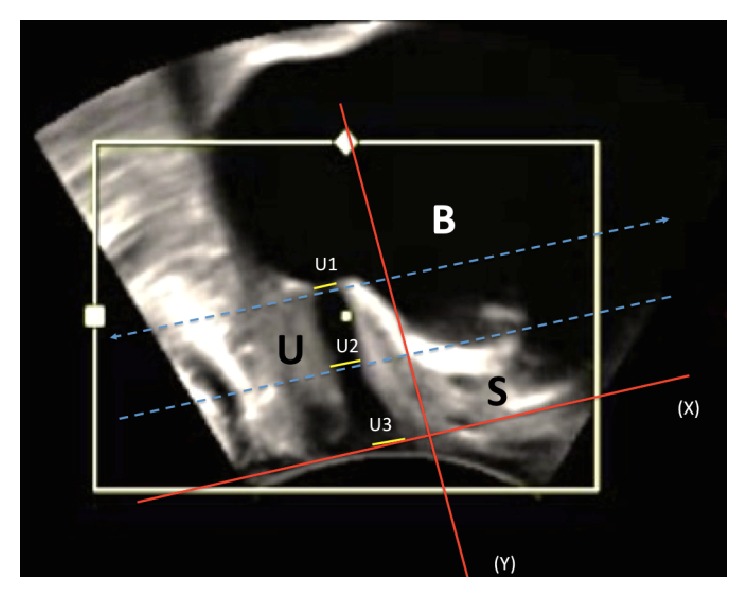
Perineal ultrasound image on midsagittal plane from a patient at rest. The positions of symphysis (S), urethra (U), bladder (B), reference line (X), the line orthogonal to reference line (Y), and urethral lumen (proximal (U1), medial (U2), and distal (U3)) are indicated.

**Figure 3:**
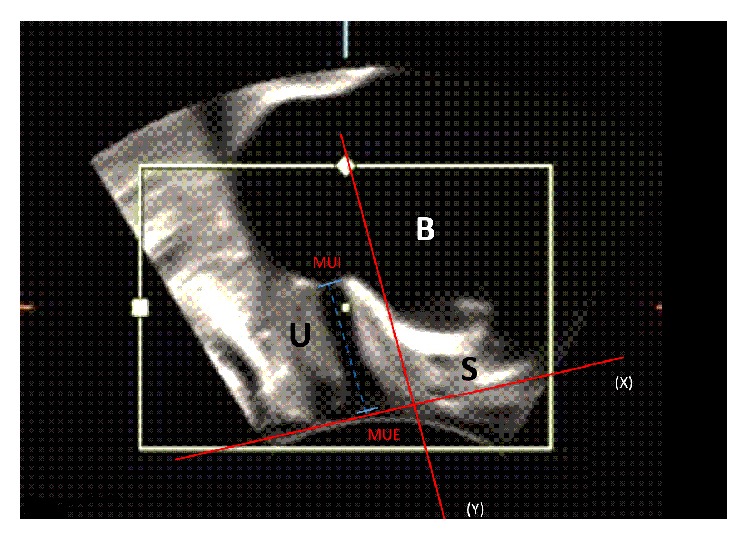
Perineal ultrasound image on midsagittal plane from a patient at rest. The positions of symphysis (S), urethra (U), bladder (B), ostium urethrae externum (MUE), meatus urethrae internum (MUI), reference line (X), and the line orthogonal to reference line (Y) are indicated. The urethral length is defined as a distance between MUE and MUI.

Transperineal ultrasound data were edited in the computer program 4D-View (GE Medical Systems). Measurements taken in a state of relaxation were used for further analysis. Three diameters of the urethral lumen in the sagittal plane were measured as shown in Figure 2: the diameter located at the ostium urethrae internum (U1), the diameter located in the middle of the urethra (U2), and the distal diameter (U3). The common problem in the transperineal ultrasound examination is the definition of the ostium urethrae externum [13]. To define the distal part of the urethra, we used a reference line according to Hennemann et al. [13]. This line is being fixed between two hyperechogenic contours of symphysis pubis. The ventral and dorsal points of the diameter were defined as a transition zone between echogenic and anechogenic parts of the urethra. The length of the urethra was measured between ostium urethrae internum and the reference line as shown in Figure 3. The urethral length (SUL) was defined as a curve between ostium urethrae internum and ostium urethrae externum, which was presented by the reference line.

The statistic program SAS Version 9.2 and Microsoft Office Excel 2007 were used for analysis. The Kruskal-Wallis test with Dunn's multiple comparisons was used. Fisher's exact test was applied for the evaluation of the urethral funneling. The results are described as statistically significant by *P* value < 0.05.

This study was performed according to the Declaration of Helsinki and approved by the local ethics committee.

## 3. Results

The 150 examined patients had a mean age of 60.4 years (range: 19–89). After examination in our outpatient urogynaecology centre, there were 41 patients with mean age of 66 years (range: 45–89) diagnosed with OAB, 67 patients with mean age 56.5 years (range: 19–80) with a diagnosis of SUI, and 42 patients in the CTRL with mean age of 61.2 years (range: 40–80).

Funneling of the bladder neck was demonstrated in 11% of the patients with the diagnosis of incontinence (Table 1) but was not found in the control group (*P* < 0.05). As presented in Table 2, we did not find any significant difference in the presence of funneling of the bladder neck between SUI and OAB (9.7% of patients with OAB and 11.9% of patients with SUI).

Mean urethral lumen U1 (Table 3) was 5.19 mm in OAB (95% CI: 4.54–5.83), 4.99 mm in SUI (95% CI: 4.47–5.52), and 4.88 mm in CTRL (95% CI: 4.46–5.30). There were no significant differences between the study groups (Table 4).

Urethral lumen U2 (Table 3) differed significantly between OAB (mean: 5.49 mm, 95% CI: 4.94–6.05) and SUI (mean: 5.17 mm, 95% CI: 4.85–5.49) when compared to CTRL (mean: 4.47 mm, 95% CI: 4.10–4.85) (*P* < 0.05). There was no significant difference in the urethral lumen U2 between OAB und SUI (Table 4).

Mean urethral lumen U3 (Table 3) did not differ significantly between the study groups (Table 4) and was 4.95 mm in OAB (95% CI: 4.21–5.69), 4.98 mm in SUI (95% CI: 4.38–5.58), and 4.96 mm in CTRL (95% CI: 4.34–5.58).

Mean urethral length (Table 3) was 2.85 cm in OAB (95% CI: 2.72–2.98), 2.82 cm in SUI (95% CI: 2.72–2.91), and 2.63 cm in CTRL (95% CI: 2.53–2.73). We have observed significant differences (*P* < 0.05) of the urethral length for OAB versus CTRL and SUI versus CTRL. There was no significant difference in the mean urethral length between OAB and SUI (Table 4).

## 4. Discussion

Transperineal ultrasound is increasingly used in the diagnostic evaluation of female urinary incontinence. Part of the ultrasound examination is to assess the urethral lumen as well as the measurement of the urethral length. The knowledge of both parameters is important for planning of incontinence surgery and for selecting an appropriate implant. In the evaluation of the urethral lumen, presence or absence of urethral funneling is the only established parameter in patients with urinary incontinence. However, no study has been done to the best of our knowledge for the evaluation of sonographically measured urethral length as well as for changes of the diameter of urethral lumen.

In contrast to current doctrines, in which it is generally believed that a shorter urethra leads to SUI, we here show for the first time by the use of 3D/4D transperineal ultrasound that the urethral length was significantly greater and the midurethral lumen was significantly wider in patients with urinary incontinence. This is best explained by structural relaxation of supportive periurethral tissue which ensures urethral tonus. However, we missed our aim to discriminate subtypes of incontinence by ultrasound assessment. Of note, we did not observe significant differences in urethral length or urethral lumen or in the presence of urethral funneling between patients with SUI and OAB. The incidence of urethral funneling, however, was significantly higher in the study groups with urinary incontinence than in the CTRL group even when mixed incontinence was excluded suggesting common pathomechanisms in both entities. This finding is interesting and merits further investigations.

Depending on the method used for assessment, mean urethral length varies from 2.78 cm to 4.1 cm [4, 14, 15]. Mitterberger et al. [15] measured urethral length by transurethral ultrasound with 3D-sonographic reconstruction and did not find any significant differences between patients with SUI and CTRL. The reason for the difference from our study could be the different ultrasound technique and small number of patients used in their study. However, together, these findings emphasize that the current doctrine of a short urethral length leading to SUI is not valid.

Our finding of a wider urethral lumen U2, actually in the middle of the urethra, in patients with SUI is in concert with observations obtained by incontinence surgery. Surgical insertion of tension-free vaginal tape (TVT) aims to stabilize the midthird of the urethra. The ideal position for TVT placement is estimated to be between 50% and 70% of the urethral length [16]. We expect that the lower urethral tonus in the middle of the urethra, according to our findings, provides the ideal function for passive support of the TVT at that part of the urethra.

The difference in the movements of the anterior and posterior walls of the proximal urethra during increase of the intraabdominal pressure causes the urethral funneling. The incidence of urethral funneling observed in women with stress urinary incontinence is reported to range from 18.6% to 97.4% [17]. A reason for the enormous variation in incidence of the urethral funneling between studies is the use of different ultrasound techniques. On the one hand, in some patients with stress urinary incontinence, urethral funneling was seen only with straining; on the other hand, some degree of urethral funneling could be already present at rest, increasing then with straining [17, 18]. Ultrasound evaluation of urethral funneling can improve diagnostics. It is one of the important qualitative parameters that can confirm the diagnosis of urinary incontinence. It is also an important parameter in the pre- and postoperative diagnostics and its presence is associated with an increased probability of therapeutic failure or recurrence [18–20].

As compared to the continent patients, the urethral lumen U2 in patients with OAB and SUI was significantly wider. The wider urethral lumen U2 can best be explained by lower urethral tonus in patients with SUI. The reason for the lower urethral tonus in SUI is a decreased volume of the rhabdosphincter as well as decreased volume of urethral smooth muscle [21]. Athanasiou et al. [22] evaluated the urethra and the urethra sphincter in women with stress urinary incontinence with 3D ultrasound and reported that urethral sphincter is thinner, smaller, and shorter in volume compared to controls. The reports on urethral vasculature are controversial. Different Doppler parameters have been studied to evaluate the vascular elements within the urethra in patients with stress urinary incontinence. Some authors [6] reported fewer periurethral vessels and Doppler flow parameters of the urethral vasculature in patients with stress urinary incontinence whereas others [7] could not find any difference in the appearance of the urethral vasculature in women with or without stress urinary incontinence. The other factors which could affect the urethral lumen are changes in the detachment of the pubourethral ligaments. Disruption of the pubourethral ligaments is significantly associated with stress urinary incontinence and has been observed in most patients with SUI by MRI studies [23, 24].

The present study reports on a new approach to guide urogynaecologists selecting patients for planned surgical strategies. Following the IDEAL recommendations [10], this refers to Stage 2a, the development level on a small collective of patients. Future prospective studies will address the therapeutic outcome in relation to the sonographic findings in a larger number of patients.

A limitation of the present study is the limited number of patients in each study group. Moreover, the CTRL consists of patients visiting our outpatient centre because of urogynaecological problems other than urinary incontinence or pelvic organ prolapse (POP-Q II or more) and hence they are not necessarily healthy women.

Strength of our study is the use of a well established and robust ultrasound technique [13, 25] that allows exact evaluation of the anatomical urethral structures with excellent repeatability and intra- or interobserver variability [3].

In conclusion, following the IDEAL recommendations [10], the reported study aimed at challenging the potential of 3D/4D sonography in the urethral morphology to provide guidance for urogynaecologists in choosing the right patient for the right procedure. According to our hypothesis, we expected differences in the urethral length as well as in the urethral lumen by patients with SUI versus CTRL. Our results have shown structural urethral changes in patients with incontinence, yet with no difference between both subtypes of urinary incontinence, SUI and OAB. Hence, anatomical findings obtained by ultrasound are still insufficient to distinguish between subtypes of urinary incontinence and cannot replace the conventional diagnostics.

## Figures and Tables

**Table 1:** Comparison of the presence of urethral funneling between women with urinary incontinence and CTRL.

Parameter	Urinary incontinence (*n* = 108)	CTRL (*n* = 42)	*P* value^*∗*^
Urethral funneling	12	0	0,02

^*∗*^Fisher's exact test, *P* value < 0.05 as significant.

**Table 2:** Comparison of the presence of urethral funneling between women with OAB and SUI.

Parameter	OAB (*n* = 41)	SUI (*n* = 67)	*P* value^*∗*^
Urethral funneling	4	8	ns

^*∗*^Fisher's exact test, *P* value < 0.05 as significant.

**Table 3:** Measurements of the urethral lumen (U1, U2, and U3) and the urethral length (SUL).

Parameter	U1 (mm)	U1 (95% CI)	U2 (mm)	U2 (95% CI)	U3 (mm)	U3 (95% CI)	SUL (cm)	SUL (95% CI)
OAB	5,19	4,54–5,83	5,49	4,94–6,05	4,95	4,21–5,69	2,85	2,72–2,98
SUI	4,99	4,47–5,52	5,17	4,85–5,49	4,98	4,38–5,58	2,82	2,72–2,91
CTRL	4,88	4,46–5,30	4,47	4,10–4,85	4,96	4,34–5,58	2,63	2,53–2,73

The results are given as mean, 95% confidence interval (CI).

**Table 4:** Comparison of the measurements of the urethral lumen (U1, U2, and U3) and urethral length (SUL) between patients with SUI and OAB and CTRL.

Parameter	*P* value (OAB versus SUI)^*∗*^	*P* value (OAB versus CTRL)^*∗*^	*P* value (SUI versus CTRL)^*∗*^
U1	ns	ns	ns
U2	ns	<0.05	<0.05
U3	ns	ns	ns
SUL	ns	<0.05	<0.05

^*∗*^Kruskal-Wallis test with Dunn's multiple comparisons, *P* value < 0.05 as significant.
